# Segmentation of Tricuspid Valve Leaflets From Transthoracic 3D Echocardiograms of Children With Hypoplastic Left Heart Syndrome Using Deep Learning

**DOI:** 10.3389/fcvm.2021.735587

**Published:** 2021-12-09

**Authors:** Christian Herz, Danielle F. Pace, Hannah H. Nam, Andras Lasso, Patrick Dinh, Maura Flynn, Alana Cianciulli, Polina Golland, Matthew A. Jolley

**Affiliations:** ^1^Children's Hospital of Philadelphia, Department of Anesthesia and Critical Care Medicine, Philadelphia, PA, United States; ^2^Computer Science and Artificial Intelligence Laboratory, Massachusetts Institute of Technology, Cambridge, MA, United States; ^3^Martinos Center for Biomedical Imaging, Massachusetts General Hospital, Harvard Medical School, Boston, MA, United States; ^4^Laboratory for Percutaneous Surgery, Queen's University, Kingston, ON, Canada; ^5^Division of Pediatric Cardiology, Children's Hospital of Philadelphia, Philadelphia, PA, United States

**Keywords:** deep learning, machine learning, image segmentation, echocardiography, tricuspid valve, congenital heart disease

## Abstract

Hypoplastic left heart syndrome (HLHS) is a severe congenital heart defect in which the right ventricle and associated tricuspid valve (TV) alone support the circulation. TV failure is thus associated with heart failure, and the outcome of TV valve repair are currently poor. 3D echocardiography (3DE) can generate high-quality images of the valve, but segmentation is necessary for precise modeling and quantification. There is currently no robust methodology for rapid TV segmentation, limiting the clinical application of these technologies to this challenging population. We utilized a Fully Convolutional Network (FCN) to segment tricuspid valves from transthoracic 3DE. We trained on 133 3DE image-segmentation pairs and validated on 28 images. We then assessed the effect of varying inputs to the FCN using Mean Boundary Distance (MBD) and Dice Similarity Coefficient (DSC). The FCN with the input of an annular curve achieved a median DSC of 0.86 [IQR: 0.81–0.88] and MBD of 0.35 [0.23–0.4] mm for the merged segmentation and an average DSC of 0.77 [0.73–0.81] and MBD of 0.6 [0.44–0.74] mm for individual TV leaflet segmentation. The addition of commissural landmarks improved individual leaflet segmentation accuracy to an MBD of 0.38 [0.3–0.46] mm. FCN-based segmentation of the tricuspid valve from transthoracic 3DE is feasible and accurate. The addition of an annular curve and commissural landmarks improved the quality of the segmentations with MBD and DSC within the range of human inter-user variability. Fast and accurate FCN-based segmentation of the tricuspid valve in HLHS may enable rapid modeling and quantification, which in the future may inform surgical planning. We are now working to deploy this network for public use.

## Introduction

Hypoplastic left heart syndrome (HLHS) is a severe congenital heart defect characterized by incomplete development of the left heart, including the mitral valve, and a left ventricle incapable of supporting the systemic circulation. HLHS would be uniformly fatal without surgical intervention, but staged surgical treatment allows children born with HLHS to survive and grow into adulthood. In contrast to a normal heart with two ventricles working in series, in HLHS, the single right ventricle (RV) remains the only functional ventricle, and the tricuspid valve (TV) is the single functional atrioventricular valve. TV failure, defined as moderate or greater regurgitation, is thus understandably associated with heart failure or death in patients with HLHS (1). Repair of the regurgitant TV in HLHS remains extremely challenging and results are suboptimal (2). Defining the mechanisms of tricuspid regurgitation (TR) is difficult, with both 2D echocardiography (2DE) and surgical inspection having significant limitations in this complex population (3). In particular, 2DE requires the echocardiographer to integrate multiple planes into a 3D mental reconstruction and to effectively convey that construction to the surgeon (4).

In contrast, transesophageal (TEE) 3D echocardiography (3DE) has transformed adult mitral valve surgery by capturing the valve's full, dynamic geometry in real time, providing an intuitive view of the functioning valve directly to the surgeon. However, clinical volume-rendering-based 3D visualization alone is insufficient to allow quantitative assessment and analysis of the valve's 3D structure or creation of physical or *in silico* models for simulation (5, 6). The development of commercially available semi-automatic 3DE-based mitral valve computer modeling tools has partially unlocked this potential, allowing precise, quantitative comparison of normal valves to dysfunctional valves, greatly informing the understanding of the 3D structural correlates of adult mitral valve dysfunction (7–10). These insights, in turn, have allowed for specific tailored surgical therapies, the development of mechanics based *in silico* modeling (6), and the simulation of patient-specific beating valve models (5). Further, 3DE-based modeling has been proven to be more accurate than 2D measurements and superior for predicting repair durability and informing surgical decision making (11). However, despite the necessity of precise information to guide complicated repairs in children and preliminary work with basic tools suggesting relationships between the 3D structure of the TV (12–14) and patient survival (15), there is no readily available methodology for the rapid 3D segmentation of the TV and, in particular, the unique valves in HLHS.

Further, valve analysis in children presents multiple practical challenges which may decrease the 3DE image quality relative to the adult population. 3DE in pediatric patients must often be acquired using transthoracic echocardiography (TTE) due to the lack of pediatric sized 3D TEE probes, which often have more artifact relative to TEE images. In addition, children are more likely to be mobile during a 3DE TTE study, and less likely to cooperate with maneuvers to increase the quality of the study, such as breath holds for high-resolution EKG-gated acquisitions. As such, studies of the TV in HLHS have primarily relied on manual segmentation or more basic measurements, all of which are subject to significant inter- and intra-user variability (12, 13, 15, 16), and are prohibitively laborious and slow for clinical use.

In the setting of these challenges, the investigation of automatic and semi-automatic methods for the segmentation of atrioventricular valves from 3DE of children has been limited (17, 18). Atlas-based segmentation has shown promising results for segmenting the adult mitral valve from TEE images and, in a small series, was demonstrated to be feasible, but less accurate for the segmentation of TV in HLHS from TTE images (19). Notably, machine learning (ML)-based approaches to the segmentation of medical images have expanded rapidly over the last decade, including applications in echocardiography (20–23), with notably increased accuracy and speed relative to other methods. In particular, the use of shape related information (landmarks, shape priors) has shown promising results (23–25). However, the application of ML-based approaches to the segmentation of atrioventricular valves (26, 27) is in its infancy. For example, the ideal inputs for a ML-based segmentation approach in a given cardiac phase are unknown. Although our aim is to use ML to segment the TV of children with HLHS in systole (TV closed), 3DE creates volume sequences which include diastole (TV open). Human segmenters often play the sequence of images to determine where the individual leaflets coapt (come together). As such, other frames may be useful as auxiliary inputs in addition to the mid-systolic (MS) frame (28). Finally, previous atlas-based studies have shown the potential for user-placed landmarks to improve the accuracy of otherwise automatic image segmentation (29–31). It is currently unknown whether such anatomical landmarks are necessary or beneficial to inform ML-based segmentation of 3DE images.

This work explores a Deep Learning (DL)-based approach using a Fully Convolutional Network (FCN) to rapidly segment the individual leaflets of the TV from TTE images of patients with HLHS. In addition, we investigate different FCN input configurations by considering combinations of various 3DE input frames and different user-provided landmarks to identify the best configuration for accurate segmentation. To the best of our knowledge, this is the first demonstration of DL-based leaflet segmentation of the TV, the first demonstration of application using TTE images, and the first application of ML to segment valves in patients with congenital heart disease.

## Materials and Methods

### Image Acquisition

In January 2016, acquisition of TTE 3DE images of the TV became part of the standard clinical echocardiography lab protocol for HLHS at the Children's Hospital of Philadelphia (14). An institutional database was utilized to retrospectively identify patients with HLHS in whom transthoracic 3DE of the TV had been previously performed. In addition, 3DE images were obtained in children with HLHS undergoing surgery as part of an institutional review board (IRB) approved research protocol. 3DE images of the TV were acquired with a field of view that captured the TV annulus and leaflets. EKG-gated, multi-beat acquisitions were obtained when patient cooperation allowed. Transthoracic X7 or X5 probes were used with the Philips IE33 and EPIQ 7 ultrasound systems (Philips Medical, Andover, MA).

In total, 161 existing TTE 3DE images in 129 unique HLHS patients were identified. Exclusion criteria included the presence of significant stitch artifacts, lack of inclusion of the entire TV and sub-valvular apparatus in the acquisition, and inability to manually delineate the TV. No patients had significant arrhythmia (including atrial fibrillation) at the time of acquisition. To complete experiments for the FCN input configurations described below, each 3DE volume sequence was required to have at least five adjacent frames before and five adjacent frames after the mid-systolic (MS) frame. Thirty-one images were from patients who were pre-stage 1 operation (median age of 4 days; IQR 3–4 days), 28 images were from patients who were post-stage 1 repair (median age of 4 months; IQR 4–5 months), 19 images were from patients who were post-stage 2 repair (median age of 34 months; IQR 18–41 months), and 83 images were from patients who were post-stage 3 repair (median age of 9 years; IQR 6–14 years). Training of the FCN was performed on 133 datasets (image volume, TV segmentation, auxiliary inputs) with pre-stage 1: 24/133 (18%), post-stage 1: 21/133 (16%), post-stage 2: 12/133 (9%), and post-stage 3: 76/133 (58%).

The remaining 28 datasets (7 at every stage of repair) were reserved for testing FCN models after training. This study was performed according to a protocol approved by the IRB at the Children's Hospital of Philadelphia.

### Ground Truth TV Segmentation

3DE images in DICOM format were imported into Philip's Qlab (Philips Medical, Andover, MA) and exported in “Cartesian DICOM” format (voxels) and then imported into 3D Slicer (32) as previously described. A trained segmenter with at least 6 months of segmenting experience selected a single mid-systolic (MS) frame for ground truth valve modeling. The segmenter reviewed the 2D and 2D color Doppler images, as well as the 3D volume-rendered images, to inform the segmentation process. In particular, review of the color Doppler images could help determine the difference between signal dropout and leaflet defect.

**Figure 1:**
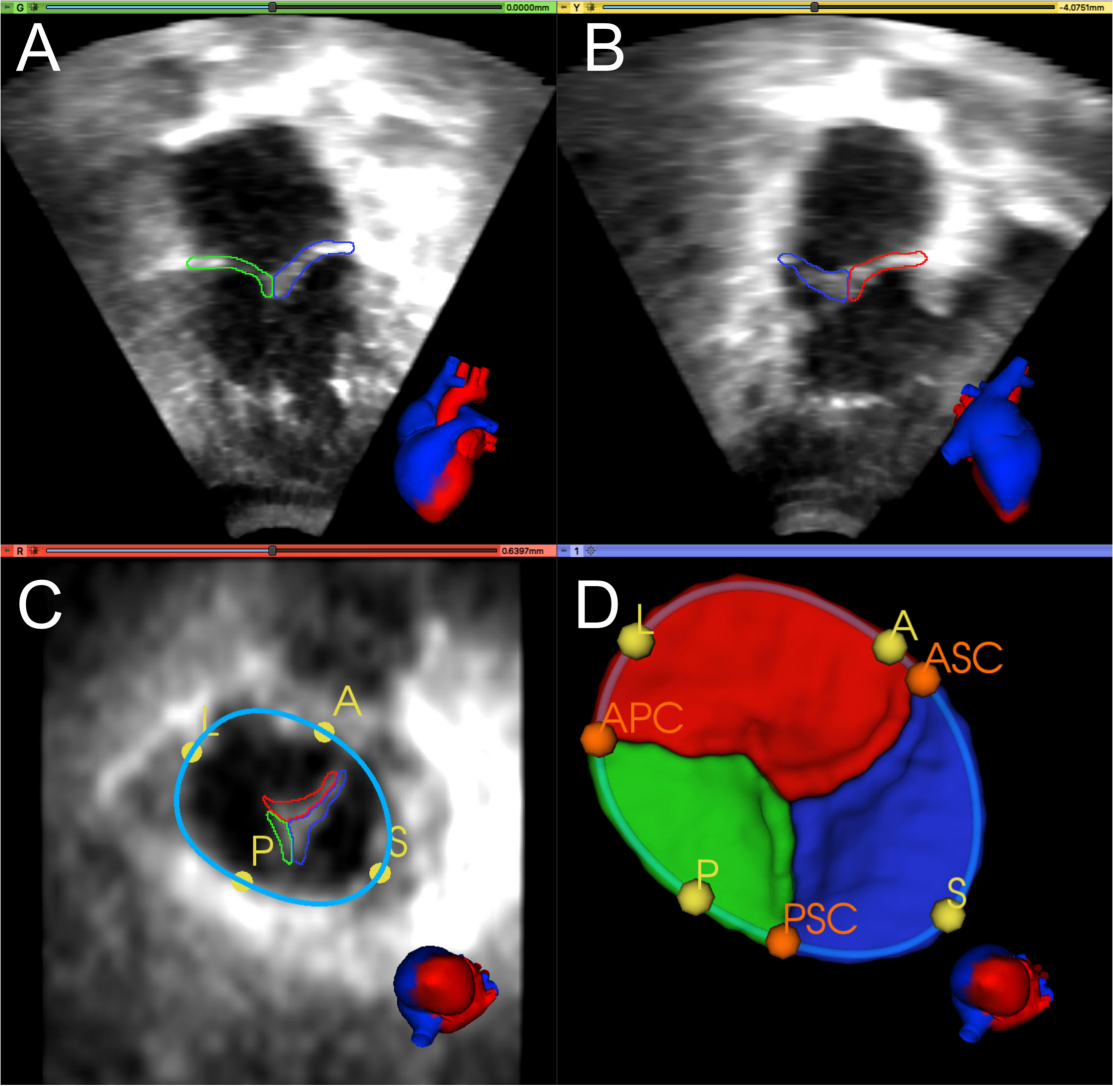
Manual segmentation and annotation of the tricuspid annulus and leaflets. **(A)** Apical 4-chamber view of 3D TTE MS frame showing the posterior (green) and septal (blue) leaflet; **(B)** Apical 2-chamber view of 3D TTE MS frame showing the anterior (red) and septal (blue) leaflet. **(C)** Ventricular view of 3D TTE MS frame with all three leaflets, the annular curve (light blue), and the quadrant landmarks (yellow): anterior (A), posterior (P), septal (S), and lateral (L). **(D)** 3D model of the segmented TV and the annular curve with APSL quadrant landmarks (yellow) and commissural landmarks (orange): anterior-septal commissure (ASC), posterior-septal commissure (PSC), and anterior-posterior commissure (APC). A heart avatar (red = left heart and aorta, blue = right heart, inferior and superior vena cava) is also provided for orientation of the echocardiographic views.

Figure 1 shows an example of TV segmentation and all annotated landmarks. First, an annular curve was manually created, as previously described (14). The annular curve creation takes an expert segmenter approximately thirty seconds for a rough curve and approximately two min for a high-precision curve. The three individual TV leaflets were manually segmented using the *SegmentEditor* module in 3D Slicer, followed by smoothing with a median filter and removal of any extraneous islands created by the manual process. Time for manual TV leaflet segmentation by an experienced segmenter varied from 2 to 4 h, depending on the complexity of the valve and quality of the image. Finally, the segmenter manually identified valve quadrant landmarks corresponding to the anterior, posterior, septal, and lateral regions (A, P, S, L) of the annulus, which took approximately 20 seconds. In addition, commissural landmarks (boundaries between the leaflets near the valve annulus) corresponding to the anterior-septal, posterior-septal, and anterior-posterior commissures (ASC, PSC, APC) were identified. These commissural landmarks were manually placed between individual leaflets by selecting points restricted to the annular curve. In this case, identification of the commissures was trivial as the leaflets had already been segmented. However, in *de novo*, unsegmented valve identification and point placement at the commissures takes approximately a minute if the annular curve has already been created, and several minutes without the guidance of an annular curve.

The inter-user and intra-user reproducibility of manual TV segmentation was assessed using images from ten HLHS subjects. To determine inter-user variability, two different expert segmenters (HN, PD) manually segmented the same 10 TVs. To assess intra-user variability, one expert segmenter (HN) segmented the same 10 TVs two times with at least one month between the first and the second segmentation process. The resulting segmentations were compared using the Dice Similarity Coefficient (DSC) and the Mean Boundary Distance (MBD), as described in the Evaluation Metrics section below.

### Data Preprocessing

As a result of differences in patient age and size, the 3DE images vary in physical voxel spacing (X: 0.21–1.02 mm, Y: 0.21–1.29 mm, Z: 0.13–0.78 mm) and image volume dimension (X: 112–288, Y: 112–304, Z: 208). Philips's Qlab software exports all volumes with the same extent of 208 voxels in Z-dimension and no resampling or resizing was applied during import to 3D Slicer. As such, we applied fully automatic image resampling and reorientation in preparation for training the FCN.

**Figure 2:**
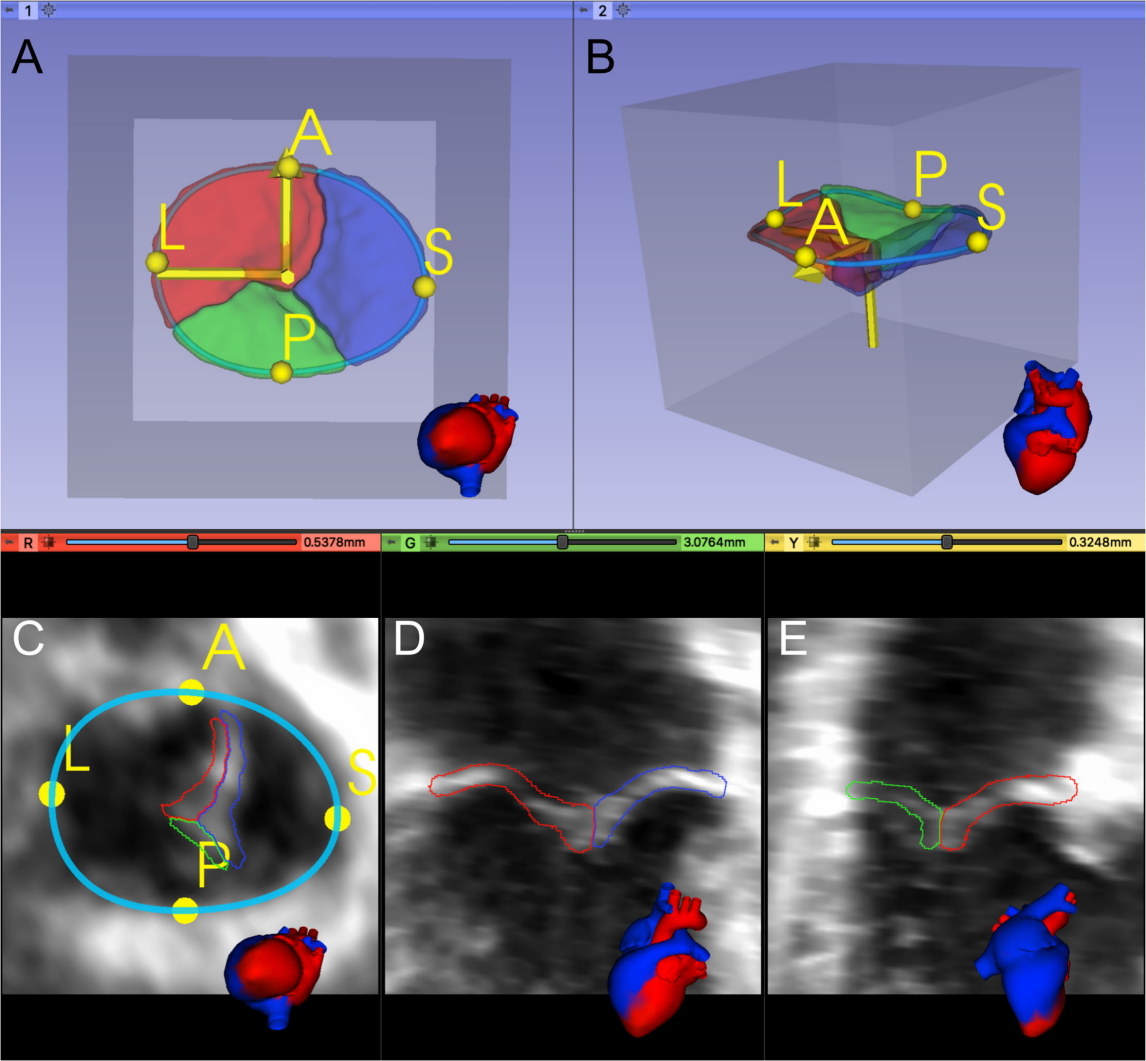
Registration and placement of the segmented TV leaflets inside a predefined Region of Interest (ROI). **(A, B)** 3D model of the segmented TV in the preprocessed data's valve coordinate system, created from the A and L landmarks and the APSL centerpoint. **(C–E)** Orthogonal 2D slices through the preprocessed data.

Considering the TV leaflets' thin structure, we performed isotropic resampling with a maximum voxel spacing of 0.25 mm while enforcing a minimum leaflet segmentation height of 6 voxels. We did so to allow sufficient spatial fidelity to represent the valve leaflets, even in the setting of transformations and resampling. If the minimum leaflet segmentation height was not met, voxel spacing was decreased accordingly. Forty three of 133 training images (32%) had a pixel spacing <0.25. In our testing datasets, twelve out of 28 images (43%) had a pixel spacing <0.25. Finally, a standard orientation was applied to each dataset using the user-defined A and L landmarks and the center point of all APSL landmarks to place it inside a region of interest (ROI) with the dimensions 224 × 224 × 224 (Figure 2).

Some of the experiments used the annular curve model, the commissural landmarks, or both as additional FCN inputs. For these experiments, the standard orientation was also applied to the annular curve model and the commissural landmarks, which were exported alongside the corresponding TV leaflet segmentations. When needed, additional frames were exported from the 3DE image sequences, as presented in the Input Frames section below.

All ground truth leaflet segmentations were visually quality checked for mislabeled leaflets by two users by plotting multiple slices of the exported segmentations providing information about a) the orientation of the valve (incorrect orientation could indicate wrong landmark placement) and b) the correct label assignment. If any mismatch was identified, it was corrected. Finally, all ground truth leaflet segmentations were automatically checked for multiple islands using the Insight Segmentation and Registration Toolkit (ITK) (33) *ConnectedComponentImageFilter*. Only the largest island was kept. Furthermore, any holes were filled using the ITK *VotingBinaryIterativeHoleFillingImageFilter*.

### Evaluation Metrics

We used two metrics to compare the ground truth segmentations to the FCN-predicted segmentations: the Dice Similarity Coefficient (DSC) (34) and the Mean Boundary Distance (MBD) (35). The DSC coefficient can be used to quantify the overlap of two segmentations (*P* and *Q*), where *DSC* = 1 indicates the segmentations are identical. The DSC is defined as follows:

$$\begin{array}{l} DSC\left(P,Q\right)=\;\frac{2|P\cap Q|}{\left|P|+|Q\right|}. \end{array}$$

Note that the DSC is very sensitive to segmentation errors for thin structures such as the TV leaflets.

The MBD is the average of bi-directional distances measured between the two segmentations (represented as surface meshes). The MBD is defined as follows:

$$\begin{array}{l} MBD=\frac{1}{2}\left(d\left(P,Q\right)+d\left(Q,P\right)\right), \end{array}$$

where *d(P,Q)* measures the mean Euclidean distance between vertex points in *P* and their closest corresponding vertices in *Q*.

We report DSC and MBD scores for (a) each of the three TV leaflets individually, (b) the average of the three TV leaflets, and (c) the valve segmentation as a single label (i.e., the individual leaflets merged into a single segmentation to allow comparison to prior published reports).

### Fully Convolutional Neural Network Architecture

**Figure 3:**
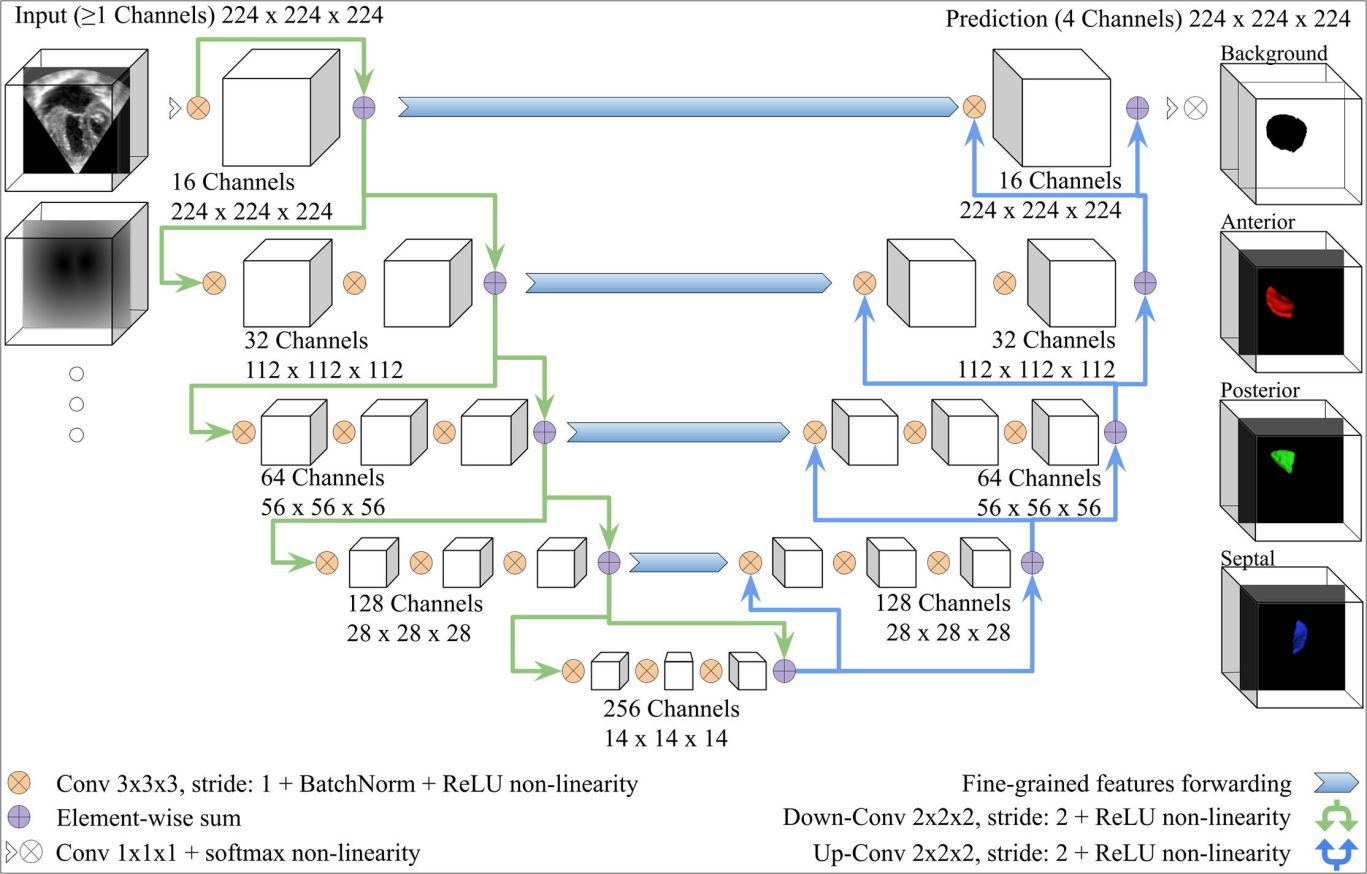
V-Net Model, modified from (34). The model input includes one channel for the MS frame and additional channels with the same dimensions for any other inputs (e.g., the annulus SDM). On the right-hand side, example 3D segmentations predicted by the FCN are displayed from the atrial view.

We adopted the V-Net architecture introduced in (34), which was inspired by the U-Net (36). Modifications were added, as shown in Figure 3. The number of FCN input channels ranged between 1 and 15, and always included the MS frame. While passing through the FCN, the number of learned filters doubled at each stage, and the dimensionality is halved, expanding the receptive field. Lastly, pixel-wise probabilities were computed by applying the softmax activation function (37) on the four-channel logits output of the FCN (three leaflets + background). We used rectified linear unit (ReLU) non-linearities instead of the Parametric ReLU (PReLU) used by the original V-Net architecture. The use of ReLU in preliminary experiments resulted in a better outcome. In order to capture the structural features of the TV, the convolution kernel size was reduced to 3 × 3 × 3 instead of 5 × 5 × 5.

### Comparison of Alternative FCN Configurations

**Figure 4:**
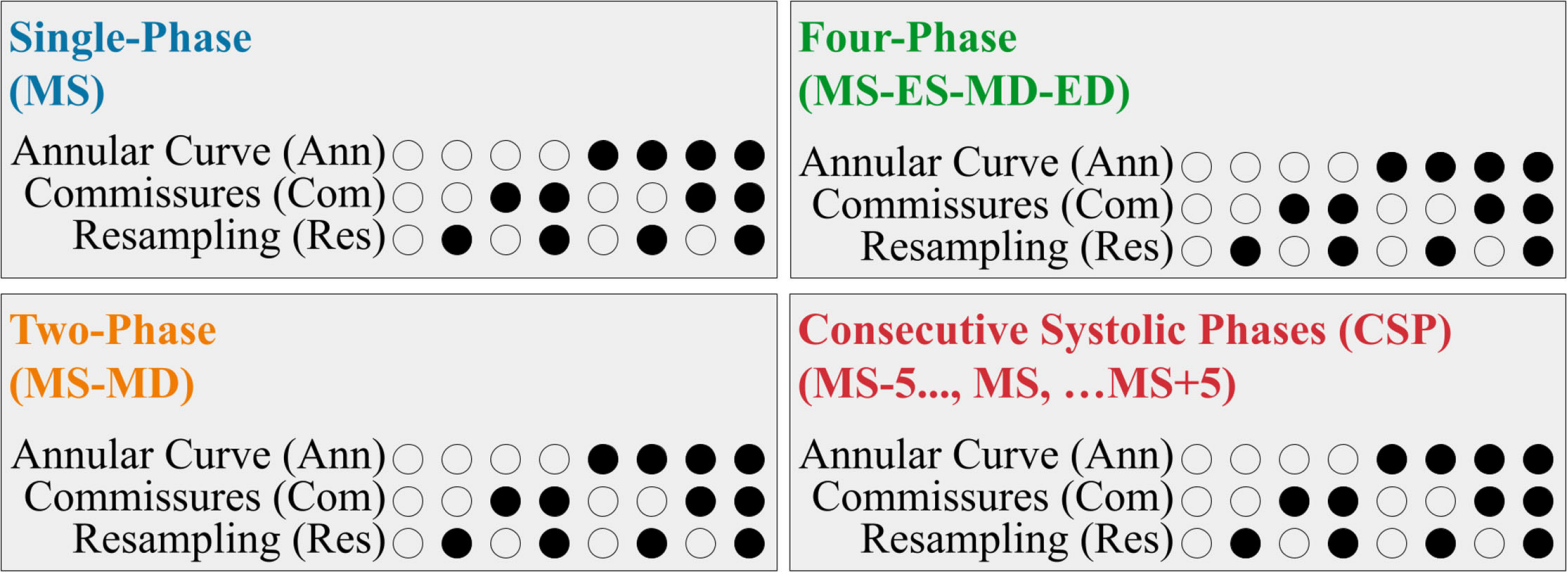
Summary of the FCN Input Variants. Each 3DE input (Single-Phase, Two-Phase, Four-Phase, and CSP) was performed with eight permutations created by the optional addition of the resampling, annular curve input, and commissural landmarks input, as depicted in the binary combination of circles (unfilled: no; filled: yes).

We evaluated the utility of several different FCN input configurations when segmenting the three individual tricuspid valve leaflets in the MS frame. A summary is shown in Figure 4.

### Input Frames

Our 3DE volume sequences contained a variable number of frames (12 to 151 frames per sequence). To assess the potential benefit of using multiple 3DE input frames on the FCN's ability to segment the targeted MS frame, we evaluated the following four input configurations:

- For the **Single-Phase** approach, only the targeted MS frame was used as the FCN input.
- The **Two-Phase** approach introduced the mid-diastolic (MD) frame in addition to the MS frame. Providing a diastolic frame (in which the TV is open) may introduce visual information not present in the MS frame to separate the valve leaflets.
- The **Four-Phase** approach added the end-systolic (ES) and end-diastolic (ED) frames, resulting in four frames (ES, ED, MS, MD) as FCN inputs.
- The **Consecutive Systolic Phases (CSP)** approach introduced the ten frames surrounding the MS frame in addition to the MS frame. The MS frame and five adjacent frames before and five adjacent frames after the MS frame were provided to the FCN. Introducing consecutive frames could provide more information regarding valve opening and closing.

### Annular Curve Input

We investigated the benefit of adding a representation of the TV annular curve as an additional FCN input. The annulus segmentation mask was converted into a signed distance map (SDM), which could be used as an additional input channel. At each voxel, SDMs store the distance to the nearest boundary voxel, thus embedding shape information into a high-dimensional space (29).

### Commissural Landmarks Input

Next, we investigated the effect of adding the commissural landmarks (ASC, APC, PSC) as additional FCN input channels. We converted all landmark coordinates into discrete spherical segmentations, each of which was converted into an SDM, as in the annular curve segmentation, resulting in three additional FCN input channels.

### Resampling

Lastly, we investigated the effect of resampling the FCN input data on the segmentation outcome. To this end, we trained each of the aforementioned FCN input configurations a) after enforcing a maximum voxel spacing and a minimum leaflet segmentation height, as described in the Data Preprocessing section above, and b) after keeping the original voxel spacing. In both cases, the 3DE volume frames were oriented and placed inside the predefined ROI.

### Data Augmentation

Geometric and intensity-based data augmentation, including random affine transformation, random contrast adjustment, and histogram clipping, was performed during model training. The random affine transformations were uniformly distributed and included rotation on any axis in range [−30, +30] degrees, translation in range [−50, 50] voxels, and scaling in range [−0.3, 0.3]. Random contrast adjustment was applied in range [−0.3, 0.3] and histogram clipping was applied at the 2nd and 99th percentile. An execution probability of 50% was used for random affine transformation, random contrast adjustment, and histogram clipping. Finally, intensity normalization was applied to the input 3DE images and any input SDMs (annular curve and/or commissural landmarks) so that all input voxels were in the range [0, 1]. We did not use elastic deformations in data augmentation as initial experiments showed no benefit.

### Learning

FCN model parameters can be learned by minimizing the empirical expected negative log-likelihood over a training dataset of images with corresponding ground truth segmentations. Often, semantic segmentation approaches only use the Soft Dice loss (34), but for thin structures like TV in 3DE images, the Soft Dice alone did not achieve satisfactory segmentation results. The assumption that the label of each voxel is conditionally independent of all other voxel labels, given the input image, leads to the conventional voxel-wise cross-entropy loss. Building upon this, we found the best results by using a loss term that is a weighted sum between a Soft Dice loss and a cross-entropy loss with spatially varying weights. We added spatially varying weights to the voxel-wise cross-entropy loss to more strongly penalize errors at voxels near the ground truth segmentation boundary, so that segmentations better snap to the borders (36, 38). In this loss, each voxel has a different weight, which can be computed as a sum between two terms: a class rebalancing weight (inverse label frequencies computed from the training segmentations, which are then normalized to sum to one) and a border weight (a constant boundary weight value ω_0_ for voxels which are less than *d*_0_ voxels from the ground truth segmentation boundary, and zero otherwise).

### Implementation Details

All methods were implemented in Python 3.7.6 using PyTorch 1.4. The NVIDIA apex library for mixed-precision training was used to speed up the training process. Model training was executed on a machine with an Intel 9940X processor simultaneously utilizing two NVIDIA Quadro RTX 8000 GPUs running CUDA 10.2 and cuDNN 7.6.5.

The final loss used for training was the weighted sum of the Soft Dice loss (34) with a weight of 1, and the earlier introduced cross-entropy loss with a weight of 0.02. We found that a higher impact factor of the boundary loss during training did not improve training results. The reason for this could be a missing basic structure in early epochs in which the boundary-aware loss results in a higher loss score. The parameters of the cross-entropy loss were ω_0_ = 50 and *d*_0_ = 3. Preliminary experiments showed that class rebalancing weights in the loss functions did not improve prediction results. For model optimization, the RAdam optimizer (39) was used which can be used for a broader range of learning rates still leading to a similar performance. We ran some preliminary testing on hyperparameters and found that a learning rate α = 0.02 was optimal. In addition, for suppression of model overfitting, we assigned a weight decay of 1e-05. All aforementioned parameters were chosen based on preliminary experiments.

The batch size varied depending on the FCN input configuration between 2 and 8. We used a learning rate scheduler that halves the learning rate after a given number of epochs (*n* = 3) have elapsed without any validation performance improvement. At training initialization, ten percent of the training datasets were reserved for validation purposes. The training process was stopped if validation performance did not improve for 30 epochs, up to a maximum of 200 epochs.

Inference used the same inputs that were used during model training. Lastly, inference results were post-processed using the ITK *ConnectedComponentImageFilter* to keep only the largest connected component for every leaflet segmentation.

### Comparison FCN Configuration and Statistical Analysis

We report the best performing FCN input configurations across individual user input groups (images only, annular curve, commissural landmarks, and the combination of annular curve and commissural landmarks). Every group includes a total of 8 FCN input configurations with different image frame inputs and with or without resampling. Within a group, each FCN was ranked based on its average leaflet MBD across individual images (*n* = 28 in all stages of repair combined) with the best FCN variant having the rank of 1 and the worst having the rank of 8. The best performing (lowest average individual leaflet MBD) FCN input configuration had the highest rank on average across all images within its group. All ranking results can be found in Supplementary Tables S17–S36.

We visualized our data and applied the Shapiro-Wilk test for normality on pairwise differences between different groups and found that our data was not normally distributed. Accordingly, we used the Wilcoxon signed-rank test for the pairwise statistical comparison of the differences in MBD and DSC across all permutations of the best FCN input configurations. Data is presented as median [IQR]. The test for normality and statistical analysis was done using the SciPy Python library 1.5.0.

## Results

**Figure 5:**
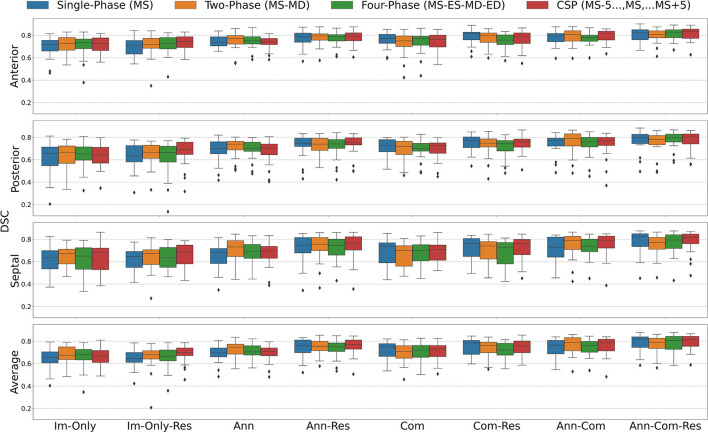
Dice Similarity Coefficient (DSC) for all stages combined for all Input Frame Combinations (Single-Phase, Two-Phase, Four-Phase and CSP) and all Binary Combinations of the Annular Curve Input (Ann), Commissural Landmarks Input (Com) and Resampling (Res). FCN input configurations using more inputs performed better than those with fewer inputs. The number of 3DE input frames suggested a less clear pattern, where more input frames did not necessarily improve the FCN output segmentations. The number of input frames did not yield a large effect on the FCN segmentations compared to the introduction of other auxiliary FCN inputs and the inclusion of resampling. Segmentation accuracy is presented for individual leaflets (anterior, posterior, septal) and the individual leaflet average. The boxes represent median DSC with IQR. The whiskers present 1.5*IQR past the low and high quartiles. The diamonds indicate outliers.

**Figure 6:**
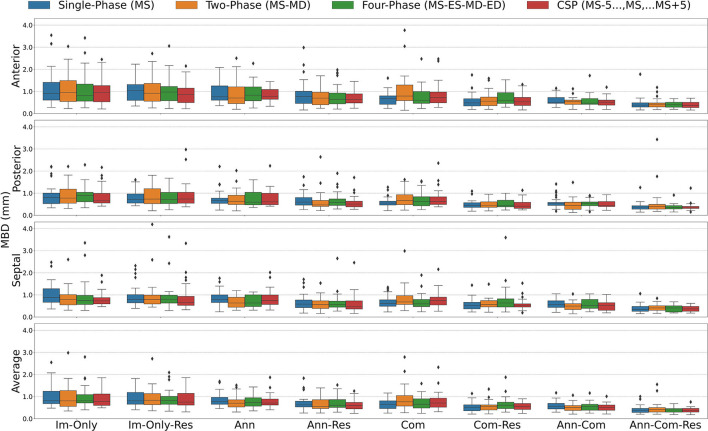
Mean Boundary Distance (MBD) for all stages combined for all Input Frame Combinations (Single-Phase, Two-Phase, Four-Phase and CSP) and all Binary Combinations of the Annular Curve Input (Ann), Commissural Landmarks Input (Com) and Resampling (Res). Segmentation accuracy is presented for individual leaflets (anterior, posterior, septal) and the individual leaflet average. The boxes represent median MBD with IQR. The whiskers present 1.5*IQR past the low and high quartiles. The diamonds indicate outliers.

The segmentation accuracy for all the FCN input configurations on the 28 unseen test images with all stages of repair combined is pictured in Figure 5 for DSC and Figure 6 for MBD and listed quantitatively in Tables 1–**4**. Additionally, the segmentation accuracy at individual stages of repair (pre-stage 1, post-stage 1, post-stage 2, post-stage 3) is pictured in Supplementary Figures S1–S4 for DSC and Supplementary Figures S5–S8 for MBD and listed quantitatively in Supplementary Tables S1–S16.

**Table 1:** Inference Results with Input Image(s) Only across all stages of repair.

		**Cropped, oriented, resampled**				**Cropped, oriented, NOT resampled**			
		**Single-Phase**	**Two-Phase**	**Four-Phase**	**CSP**	**Single-Phase**	**Two-Phase**	**Four–Phase**	**CSP**
DSC	Anterior	0.71[0.63–0.75]	0.72[0.68–0.77]	0.73[0.68–0.78]	0.74[0.69–0.79]	0.72[0.66–0.76]	0.73[0.67–0.78]	0.74[0.68–0.77]	**0.73[0.66–0.78]**
	Posterior	0.63[0.58–0.73]	0.66[0.61–0.73]	0.66[0.58–0.72]	0.69[0.65–0.76]	0.66[0.55–0.71]	0.67[0.57–0.72]	0.65[0.6–0.71]	**0.64[0.57–0.71]**
	Septal	0.65[0.55–0.69]	0.67[0.57–0.71]	0.64[0.55–0.73]	0.69[0.58–0.75]	0.64[0.54–0.7]	0.67[0.58–0.71]	0.65[0.53–0.72]	**0.68[0.53–0.72]**
	Merged	0.75[0.7–0.78]	0.77[0.73–0.8]	0.76[0.73–0.8]	0.77[0.72–0.81]	0.74[0.7–0.79]	0.77[0.73–0.81]	0.76[0.71–0.81]	**0.76[0.71–0.8]**
	Average	0.64[0.61–0.7]	0.68[0.64–0.72]	0.67[0.63–0.72]	0.7[0.67–0.74]	0.65[0.61–0.7]	0.67[0.64–0.75]	0.68[0.63–0.73]	**0.67[0.61–0.72]**
MBD	Anterior	1.03[0.6–1.31]	0.92[0.55–1.36]	0.97[0.58–1.22]	0.85[0.49–1.15]	0.91[0.61–1.41]	0.95[0.55–1.49]	0.81[0.56–1.33]	**0.96[0.53–1.26]**
	Posterior	0.72[0.55–0.97]	0.73[0.53–1.2]	0.72[0.53–1.03]	0.74[0.55–1.04]	0.81[0.53–1]	0.77[0.54–1.18]	0.88[0.61–1.04]	**0.66[0.56–0.99]**
	Septal	0.79[0.64–1.01]	0.79[0.59–0.99]	0.8[0.63–0.99]	0.65[0.52–0.93]	0.88[0.66–1.28]	0.79[0.55–1.01]	0.75[0.57–0.98]	**0.73[0.59–0.87]**
	Merged	0.67[0.56–0.82]	0.6[0.46–0.69]	0.62[0.52–0.81]	0.59[0.47–0.79]	0.72[0.58–1.09]	0.66[0.49–0.88]	0.65[0.49–0.9]	**0.61[0.5–0.79]**
	Average	0.81[0.66–1.19]	0.84[0.64–1.14]	0.83[0.65–1.01]	0.75[0.61–1.14]	0.82[0.68–1.24]	0.81[0.56–1.27]	0.81[0.71–1.09]	**0.78[0.6–1.12]**

*DSC and MBD measurements are presented for individual leaflets (anterior, posterior, septal), merged leaflets and average across individual leaflets. The columns represent different image frame inputs with and without resampling. The column of the best ranked FCN input configuration in bold. Metric values are displayed as median [IQR]*.

### FCN Segmentation Using Input Frames Only

For all stages combined, when the images alone were provided, the consecutive systolic phases (CSP) FCN input configuration without resampling had the highest rank with the lowest MBD on average (Table 1). However, the highest rank FCN varied across images of patients at various stages of repair. For post stage 3 images, the highest ranked FCN was the same as for the whole population as noted above. However, at post-stage 1 and post-stage 2, the CSP FCN variant with resampling performed superiorly across the whole group. At pre-stage 1, the Two-Phase FCN variant ranked best. Across all user input groups, the best performing FCN variants of post-stage 3 repair and all stages of repair combined were the only FCN variants performing superior without resampling.

### FCN Segmentation Using the Annular Curve

After adding the annular curve as an input, for all stages combined and individual stages (post-repair 1, post-repair 2), the CSP FCN input configuration with resampling had the highest rank with the highest DSC and lowest MBD on average. For pre-stage 1 and post-stage 3, the Two-Phase FCN variant ranked highest (Table 2).

**Table 2:** Inference Results Using Annular Curve Input across all stages of repair.

		**Cropped, oriented, resampled**				**Cropped, oriented, NOT resampled**			
		**Single-Phase**	**Two-Phase**	**Four-Phase**	**CSP**	**Single-Phase**	**Two-Phase**	**Four–Phase**	**CSP**
DSC	Anterior	0.79[0.74–0.82]	0.79[0.76–0.81]	0.79[0.75–0.81]	**0.79[0.75–0.82]**	0.74[0.71–0.79]	0.77[0.72–0.8]	0.75[0.72–0.79]	0.74[0.72–0.77]
	Posterior	0.74[0.72–0.79]	0.74[0.68–0.79]	0.74[0.7–0.78]	**0.75[0.73–0.8]**	0.7[0.65–0.76]	0.74[0.69–0.76]	0.7[0.67–0.76]	0.7[0.64–0.74]
	Septal	0.75[0.68–0.82]	0.76[0.7–0.82]	0.74[0.66–0.8]	**0.77[0.7–0.82]**	0.68[0.58–0.72]	0.73[0.65–0.79]	0.69[0.63–0.75]	0.7[0.63–0.74]
	Merged	0.86[0.8–0.87]	0.85[0.8–0.88]	0.84[0.8–0.86]	**0.86[0.81–0.88]**	0.81[0.77–0.83]	0.83[0.8–0.85]	0.81[0.77–0.84]	0.8[0.74–0.82]
	Average	0.76[0.7–0.81]	0.75[0.72–0.8]	0.75[0.71–0.78]	**0.77[0.73–0.81]**	0.7[0.66–0.74]	0.75[0.69–0.77]	0.7[0.68–0.76]	0.71[0.67–0.74]
MBD	Anterior	0.77[0.46–1.01]	0.7[0.4–0.96]	0.64[0.44–0.93]	**0.63[0.48–0.89]**	0.75[0.61–1.25]	0.7[0.45–1.21]	0.83[0.58–1.2]	0.78[0.64–1.09]
	Posterior	0.59[0.46–0.8]	0.47[0.4–0.67]	0.59[0.45–0.74]	**0.5[0.38–0.63]**	0.68[0.54–0.77]	0.63[0.49–0.9]	0.59[0.47–1.04]	0.6[0.48–0.99]
	Septal	0.58[0.43–0.77]	0.57[0.38–0.73]	0.56[0.44–0.71]	**0.46[0.36–0.73]**	0.8[0.65–1]	0.63[0.44–0.89]	0.63[0.47–1]	0.74[0.57–1]
	Merged	0.34[0.26–0.39]	0.33[0.24–0.39]	0.38[0.29–0.44]	**0.35[0.23–0.4]**	0.48[0.38–0.58]	0.49[0.38–0.62]	0.44[0.28–0.57]	0.55[0.46–0.68]
	Average	0.62[0.53–0.79]	0.55[0.46–0.86]	0.61[0.51–0.86]	**0.6[0.44–0.74]**	0.78[0.65–0.97]	0.7[0.53–0.86]	0.72[0.57–0.95]	0.71[0.62–0.89]

*DSC and MBD measurements are presented for individual leaflets (anterior, posterior, septal), merged leaflets and average across individual leaflets. The columns represent different image frame inputs with and without resampling. The column of the best ranked FCN input configuration in bold. Metric values are displayed as median [IQR]*.

### FCN Segmentation Using the Commissural Landmarks

When the commissural landmarks formed the only auxiliary inputs, for all stages combined and individual stages (pre-stage 1, post-stage 1), the Single-Phase FCN input configuration with resampling had the highest rank with the lowest MBD on average (Table 3). For post-stage 2 repair and post-stage 3 repair, the Two-Phase FCN variant and the CSP FCN variant, respectively, ranked highest.

**Table 3:** Inference Results Using the Commissural Landmarks across all stages of repair.

		**Cropped, oriented, resampled**				**Cropped, oriented, NOT resampled**			
		**Single-Phase**	**Two-Phase**	**Four-Phase**	**CSP**	**Single-Phase**	**Two-Phase**	**Four–Phase**	**CSP**
DSC	Anterior	**0.81[0.76–0.83]**	0.8[0.74–0.82]	0.76[0.72–0.8]	0.79[0.73–0.82]	0.77[0.73–0.81]	0.75[0.7–0.79]	0.77[0.71–0.79]	0.76[0.7–0.8]
	Posterior	**0.77[0.71–0.8]**	0.75[0.71–0.78]	0.74[0.68–0.77]	0.76[0.71–0.78]	0.73[0.67–0.78]	0.72[0.65–0.76]	0.7[0.68–0.75]	0.73[0.66–0.75]
	Septal	**0.76[0.65–0.81]**	0.74[0.62–0.78]	0.73[0.58–0.77]	0.76[0.66–0.8]	0.73[0.59–0.77]	0.7[0.56–0.74]	0.7[0.61–0.75]	0.71[0.61–0.75]
	Merged	**0.82[0.75–0.84]**	0.81[0.75–0.83]	0.8[0.74–0.83]	0.81[0.77–0.84]	0.8[0.75–0.83]	0.78[0.74–0.81]	0.79[0.73–0.82]	0.79[0.73–0.81]
	Average	**0.78[0.68–0.81]**	0.76[0.7–0.79]	0.72[0.68–0.78]	0.76[0.7–0.8]	0.75[0.66–0.78]	0.71[0.65–0.76]	0.72[0.66–0.76]	0.73[0.66–0.76]
MBD	Anterior	**0.48[0.34–0.67]**	0.56[0.36–0.74]	0.6[0.46–0.94]	0.53[0.37–0.73]	0.67[0.42–0.8]	0.79[0.59–1.29]	0.6[0.46–1]	0.72[0.48–0.97]
	Posterior	**0.45[0.35–0.57]**	0.43[0.36–0.61]	0.46[0.38–0.68]	0.41[0.31–0.58]	0.55[0.46–0.63]	0.67[0.48–0.93]	0.62[0.43–0.84]	0.63[0.49–0.83]
	Septal	**0.51[0.37–0.67]**	0.57[0.46–0.74]	0.63[0.45–0.82]	0.53[0.45–0.59]	0.61[0.49–0.77]	0.68[0.57–0.96]	0.59[0.47–0.79]	0.75[0.55–0.9]
	Merged	**0.49[0.4–0.63]**	0.5[0.41–0.61]	0.46[0.39–0.66]	0.47[0.37–0.65]	0.59[0.45–0.81]	0.65[0.46–0.91]	0.58[0.49–0.8]	0.6[0.48–0.84]
	Average	**0.52[0.36–0.63]**	0.56[0.4–0.63]	0.61[0.46–0.75]	0.54[0.42–0.69]	0.64[0.46–0.81]	0.77[0.58–1.05]	0.65[0.48–0.9]	0.71[0.52–0.93]

*DSC and MBD measurements are presented for individual leaflets (anterior, posterior, septal), merged leaflets and average across individual leaflets. The columns represent different image frame inputs with and without resampling. The column of the best ranked FCN input configuration in bold. Metric values are displayed as median [IQR]*.

### FCN Segmentation Using the Annular Curve and Commissural Landmarks

When the annular curve and commissural landmarks combined were used, for all stages combined and individual stages (post-stage 1, post-stage 2) the CSP FCN input configuration with resampling had the highest rank with the lowest MBD on average (Table 4). For pre-stage 1 and post-stage 3, the Single-Phase FCN variant ranked highest.

**Table 4:** Inference results using annular curve input and commissural landmarks across all stages of repair.

		**Cropped, oriented, resampled**				**Cropped, oriented, NOT resampled**			
		**Single-Phase**	**Two-Phase**	**Four-Phase**	**CSP**	**Single-Phase**	**Two-Phase**	**Four–Phase**	**CSP**
DSC	Anterior	0.82[0.76–0.85]	0.81[0.78–0.84]	0.83[0.78–0.85]	**0.84[0.77–0.86]**	0.8[0.74–0.82]	0.81[0.75–0.84]	0.78[0.75–0.8]	0.81[0.76–0.84]
	Posterior	0.8[0.74–0.83]	0.78[0.73–0.82]	0.8[0.76–0.83]	**0.8[0.74–0.83]**	0.77[0.72–0.79]	0.79[0.72–0.83]	0.76[0.71–0.8]	0.77[0.73–0.8]
	Septal	0.82[0.73–0.85]	0.77[0.71–0.82]	0.79[0.72–0.84]	**0.82[0.76–0.85]**	0.73[0.64–0.82]	0.79[0.71–0.83]	0.74[0.69–0.8]	0.79[0.72–0.83]
	Merged	0.86[0.81–0.88]	0.85[0.8–0.87]	0.86[0.81–0.87]	**0.85[0.82–0.88]**	0.83[0.79–0.86]	0.84[0.79–0.87]	0.82[0.79–0.85]	0.84[0.8–0.86]
	Average	0.82[0.74–0.84]	0.79[0.74–0.83]	0.81[0.73–0.84]	**0.81[0.75–0.84]**	0.76[0.69–0.81]	0.79[0.71–0.83]	0.76[0.7–0.8]	0.79[0.71–0.82]
MBD	Anterior	0.38[0.29–0.48]	0.42[0.3–0.48]	0.39[0.28–0.51]	**0.36[0.27–0.51]**	0.5[0.46–0.72]	0.54[0.41–0.61]	0.47[0.42–0.68]	0.51[0.38–0.61]
	Posterior	0.37[0.28–0.43]	0.4[0.28–0.49]	0.35[0.28–0.44]	**0.37[0.31–0.4]**	0.52[0.45–0.58]	0.45[0.27–0.59]	0.53[0.43–0.6]	0.44[0.39–0.62]
	Septal	0.34[0.26–0.48]	0.41[0.3–0.51]	0.41[0.23–0.51]	**0.37[0.26–0.48]**	0.57[0.43–0.73]	0.49[0.34–0.6]	0.51[0.39–0.79]	0.52[0.3–0.64]
	Merged	0.34[0.25–0.4]	0.36[0.24–0.42]	0.35[0.21–0.4]	**0.33[0.26–0.4]**	0.47[0.34–0.55]	0.43[0.29–0.64]	0.44[0.35–0.56]	0.43[0.31–0.5]
	Average	0.38[0.28–0.46]	0.42[0.3–0.5]	0.4[0.3–0.46]	**0.38[0.3–0.46]**	0.56[0.44–0.69]	0.51[0.37–0.59]	0.56[0.39–0.65]	0.5[0.38–0.62]

*DSC and MBD measurements are presented for individual leaflets (anterior, posterior, septal), merged leaflets and average across individual leaflets. The columns represent different image frame inputs with and without resampling. The column of the best ranked FCN input configuration in bold. Metric values are displayed as median [IQR]*.

### Comparison of FCN Inference Results to Reproducibility of Manual Segmentation

Table 5 compares FCN segmentation accuracy metrics for the best input configurations to each other and to those of expert human segmenters. In particular, the FCN using the annular curve and commissural landmarks as inputs was superior to the same expert segmenter repeating a given segmentation on average. When using the annulus only or the commissures only, the FCN performed nearly equivalently to the same expert segmenter repeating a given segmentation, and superiorly to the difference between two experts segmenting the same image. FCN input of images alone resulted in a somewhat lower DSC and greater MBD than the measurements of inter- and intra-rater variability.

**Table 5:** Comparison of the best ranked inference results across all stages of repair to expert segmentation.

	**Input Config**	**FCN**				**Expert**	
		**Images Only**	**Annulus**	**Commissures**	**Annulus + Commissures**	**Inter–User**	**Intra–User**
	**Input Frames**	**CSP**	**CSP**	**Single-Phase**	**CSP**		
	**Resampling**	**No**	**Yes**	**Yes**	**Yes**	**N/A**	**N/A**
DSC	Anterior	0.73[0.66–0.78]	0.79[0.75–0.82]	0.81[0.76–0.83]	0.84[0.77–0.86]	0.78[0.75–0.8]	0.84[0.83–0.86]
	Posterior	0.64[0.57–0.71]	0.75[0.73–0.8]	0.77[0.71–0.8]	0.8[0.74–0.83]	0.73[0.69–0.76]	0.77[0.71–0.8]
	Septal	0.68[0.53–0.72]	0.77[0.7–0.82]	0.76[0.65–0.81]	0.82[0.76–0.85]	0.72[0.68–0.78]	0.84[0.79–0.85]
	Merged	0.76[0.71–0.8]	0.86[0.81–0.88]	0.82[0.75–0.84]	0.85[0.82–0.88]	0.81[0.79–0.84]	0.87[0.85–0.87]
	Average	0.67[0.61–0.72]	0.77[0.73–0.81]	0.78[0.68–0.81]	0.81[0.75–0.84]	0.74[0.7–0.78]	0.82[0.79–0.83]
MBD	Anterior	0.96[0.53–1.26]	0.63[0.48–0.89]	0.48[0.34–0.67]	0.36[0.27–0.51]	0.83[0.52–1.05]	0.49[0.38–0.54]
	Posterior	0.66[0.56–0.99]	0.5[0.38–0.63]	0.45[0.35–0.57]	0.37[0.31–0.4]	0.93[0.82–1.17]	0.59[0.46–0.7]
	Septal	0.73[0.59–0.87]	0.46[0.36–0.73]	0.51[0.37–0.67]	0.37[0.26–0.48]	0.92[0.64–0.97]	0.62[0.41–1.07]
	Merged	0.61[0.5–0.79]	0.35[0.23–0.4]	0.49[0.4–0.63]	0.33[0.26–0.4]	0.88[0.63–1.03]	0.39[0.32–0.49]
	Average	0.78[0.6–1.12]	0.6[0.44–0.74]	0.52[0.36–0.63]	0.38[0.3–0.46]	0.92[0.77–0.96]	0.55[0.46–0.69]

*DSC and MBD measurements are presented for individual leaflets (anterior, posterior, septal), merged leaflets and average across individual leaflets. All metric values are displayed as median [IQR]. The best ranked FCN input configuration across individual user input groups are displayed on the left and the expert segmenter results (inter-user and intra-user) are shown on the right. For individual leaflet averages, with providing more user input, the FCN results gradually improve. When providing the annulus contour, the FCN performs superior to the inter–user and is on par with the intra-user segmentation results. When providing the annulus contour and commissural landmarks combined, the FCN outperforms expert segmentation*.

### Comparison of FCN

The median DSC, median MBD, and corresponding *p*-values for merged leaflets and averages across individual leaflets are shown in Tables 6, 7, respectively. These values are provided for individual stages of repair and for all stages of repair combined.

**Table 6:** Median DSC and Median MBD of merged leaflets, and *p* values for two–sided non–parametric Wilcoxon signed–rank test at individual stages and all stages combined.

	**FCN Configuration A**	**FCN Configuration B**	**Med. DSC A**	**Med. DSC B**	***p Value***	**Med. MBD A**	**Med. MBD B**	***p Value***
Pre–Stage1	(Two-Phase) Im-Only-Res	(Two-Phase) Ann-Res	0.80 [0.77–0.81]	0.88 [0.87–0.89]	0.02*	0.44 [0.41–0.47]	0.19 [0.17–0.23]	0.02*
	(Two-Phase) Im-Only-Res	(Single-Phase) Com-Res	0.80 [0.77–0.81]	0.85 [0.84–0.86]	0.02*	0.44 [0.41–0.47]	0.39 [0.27–0.43]	0.22
	(Two-Phase) Im-Only-Res	(Single-Phase) Ann-Com-Res	0.80 [0.77–0.81]	0.89 [0.87–0.89]	0.02*	0.44 [0.41–0.47]	0.18 [0.16–0.21]	0.02*
	(Two-Phase) Ann-Res	(Single-Phase) Com-Res	0.88 [0.87–0.89]	0.85 [0.84–0.86]	0.02*	0.19 [0.17–0.23]	0.39 [0.27–0.43]	0.05*
	(Two-Phase) Ann-Res	(Single-Phase) Ann-Com-Res	0.88 [0.87–0.89]	0.89 [0.87–0.89]	0.94	0.19 [0.17–0.23]	0.18 [0.16–0.21]	0.16
	(Single-Phase) Com-Res	(Single-Phase) Ann-Com-Res	0.85 [0.84–0.86]	0.89 [0.87–0.89]	0.02*	0.39 [0.27–0.43]	0.18 [0.16–0.21]	0.05*
Post–Stage1	(CSP) Im-Only-Res	(CSP) Ann-Res	0.70 [0.68–0.75]	0.83 [0.78–0.84]	0.03*	0.66 [0.45–0.84]	0.35 [0.26–0.38]	0.02*
	(CSP) Im-Only-Res	(Single-Phase) Com-Res	0.70 [0.68–0.75]	0.75 [0.74–0.76]	0.03*	0.66 [0.45–0.84]	0.54 [0.45–0.63]	0.08
	(CSP) Im-Only-Res	(CSP) Ann-Com-Res	0.70 [0.68–0.75]	0.83 [0.76–0.85]	0.02*	0.66 [0.45–0.84]	0.40 [0.33–0.42]	0.02*
	(CSP) Ann-Res	(Single-Phase) Com-Res	0.83 [0.78–0.84]	0.75 [0.74–0.76]	0.11	0.35 [0.26–0.38]	0.54 [0.45–0.63]	0.05*
	(CSP) Ann-Res	(CSP) Ann-Com-Res	0.83 [0.78–0.84]	0.83 [0.76–0.85]	0.47	0.35 [0.26–0.38]	0.40 [0.33–0.42]	0.22
	(Single-Phase) Com-Res	(CSP) Ann-Com-Res	0.75 [0.74–0.76]	0.83 [0.76–0.85]	0.08	0.54 [0.45–0.63]	0.40 [0.33–0.42]	0.08
Post–Stage2	(CSP) Im-Only-Res	(CSP) Ann-Res	0.75 [0.74–0.78]	0.81 [0.77–0.83]	0.02*	0.57 [0.54–0.73]	0.37 [0.36–0.47]	0.03*
	(CSP) Im-Only-Res	(Two-Phase) Com-Res	0.75 [0.74–0.78]	0.76 [0.75–0.80]	0.08	0.57 [0.54–0.73]	0.54 [0.47–0.57]	0.05*
	(CSP) Im-Only-Res	(CSP) Ann-Com-Res	0.75 [0.74–0.78]	0.82 [0.79–0.84]	0.02*	0.57 [0.54–0.73]	0.34 [0.33–0.40]	0.02*
	(CSP) Ann-Res	(Two-Phase) Com-Res	0.81 [0.77–0.83]	0.76 [0.75–0.80]	0.02*	0.37 [0.36–0.47]	0.54 [0.47–0.57]	0.05*
	(CSP) Ann-Res	(CSP) Ann-Com-Res	0.81 [0.77–0.83]	0.82 [0.79–0.84]	0.11	0.37 [0.36–0.47]	0.34 [0.33–0.40]	0.02*
	(Two-Phase) Com-Res	(CSP) Ann-Com-Res	0.76 [0.75–0.80]	0.82 [0.79–0.84]	0.02*	0.54 [0.47–0.57]	0.34 [0.33–0.40]	0.03*
Post–Stage3	(CSP) Im-Only	(Two-Phase) Ann-Res	0.80 [0.76–0.82]	0.87 [0.86–0.88]	0.02*	0.61 [0.47–1.37]	0.34 [0.30–0.37]	0.02*
	(CSP) Im-Only	(CSP) Com-Res	0.80 [0.76–0.82]	0.84 [0.82–0.85]	0.22	0.61 [0.47–1.37]	0.58 [0.47–0.67]	0.38
	(CSP) Im-only	(Single-Phase) Ann-Com-Res	0.80 [0.76–0.82]	0.88 [0.87–0.88]	0.02*	0.61 [0.47–1.37]	0.33 [0.32–0.37]	0.02*
	(Two-Phase) Ann-Res	(CSP) Com-Res	0.87 [0.86–0.88]	0.84 [0.82–0.85]	0.02*	0.34 [0.30–0.37]	0.58 [0.47–0.67]	0.03*
	(Two-Phase) Ann-Res	(Single-Phase) Ann-Com-Res	0.87 [0.86–0.88]	0.88 [0.87–0.88]	0.81	0.34 [0.30–0.37]	0.33 [0.32–0.37]	0.94
	(CSP) Com-Res	(Single-Phase) Ann-Com-Res	0.84 [0.82–0.85]	0.88 [0.87–0.88]	0.02*	0.58 [0.47–0.67]	0.33 [0.32–0.37]	0.02*
All Stages	(CSP) Im-Only	(CSP) Ann-Res	0.76 [0.71–0.80]	0.86 [0.81–0.88]	<0.001*	0.61 [0.50–0.79]	0.35 [0.23–0.40]	<0.001*
	(CSP) Im-Only	(Single-Phase) Com-Res	0.76 [0.71–0.80]	0.82 [0.75–0.84]	<0.001*	0.61 [0.50–0.79]	0.49 [0.40–0.63]	0.004*
	(CSP) Im-Only	(CSP) Ann-Com-Res	0.76 [0.71–0.80]	0.85 [0.82–0.88]	<0.001*	0.61 [0.50–0.79]	0.33 [0.26–0.40]	<0.001*
	(CSP) Ann-Res	(Single-Phase) Com-Res	0.86 [0.81–0.88]	0.82 [0.75–0.84]	<0.001*	0.35 [0.23–0.40]	0.49 [0.40–0.63]	<0.001*
	(CSP) Ann-Res	(CSP) Ann-Com-Res	0.86 [0.81–0.88]	0.85 [0.82–0.88]	0.09	0.35 [0.23–0.40]	0.33 [0.26–0.40]	0.49
	(Single-Phase) Com-Res	(CSP) Ann-Com-Res	0.82 [0.75–0.84]	0.85 [0.82–0.88]	<0.001*	0.49 [0.40–0.63]	0.33 [0.26–0.40]	<0.001*

The best ranked FCN input configurations across individual user inputs were compared. Different stages are presented along the larger row blocks. Metric values are displayed as median [IQR]. No additional user inputs (Im-Only), annular curve input (Ann), commissural landmarks input (Com) and resampling (Res). Significant p values (<0.05) marked with an asteroid (*). Significant differences could be found in almost all pairwise combinations except when comparing the use of input images only with commissural landmarks input only and when comparing the annular curve input only and the annular curve combined with the commissural landmarks. In addition, for post–stage 1 repair, no significant differences could be found when comparing annular curve input only with commissural landmarks input only and when comparing commissural landmarks only with the combination of annular curve and commissural landmarks.

**Table 7:** Median DSC and MBD of the average across leaflets, and *p* values for two–sided non–parametric Wilcoxon signed–rank test at individual stages and all stages combined.

	**FCN Configuration A**	**FCN Configuration B**	**Med. DSC A**	**Med. DSC B**	***p Value***	**Med. MBD A**	**Med. MBD B**	***p Value***
Pre–Stage1	(Two-Phase) Im-Only-Res	(Two-Phase) Ann-Res	0.72[0.69–0.76]	0.81[0.76–0.83]	0.02*	0.59[0.49–0.66]	0.41[0.34–0.48]	0.03*
	(Two-Phase) Im-Only-Res	(Single-Phase) Com-Res	0.72[0.69–0.76]	0.81[0.80–0.81]	0.02*	0.59[0.49–0.66]	0.30[0.26–0.33]	0.02*
	(Two-Phase) Im-Only-Res	(Single-Phase) Ann-Com-Res	0.72[0.69–0.76]	0.85[0.82–0.86]	0.02*	0.59[0.49–0.66]	0.21[0.21–0.27]	0.02*
	(Two-Phase) Ann-Res	(Single-Phase) Com-Res	0.81[0.76–0.83]	0.81[0.80–0.81]	0.81	0.41[0.34–0.48]	0.30[0.26–0.33]	0.08
	(Two-Phase) Ann-Res	(Single-Phase) Ann-Com-Res	0.81[0.76–0.83]	0.85[0.82–0.86]	0.02*	0.41[0.34–0.48]	0.21[0.21–0.27]	0.03*
	(Single-Phase) Com-Res	(Single-Phase) Ann-Com-Res	0.81[0.80–0.81]	0.85[0.82–0.86]	0.02*	0.30[0.26–0.33]	0.21[0.21–0.27]	0.02*
Post–Stage1	(CSP) Im-Only-Res	(CSP) Ann-Res	0.59[0.57–0.69]	0.74[0.65–0.79]	0.08	0.63[0.48–1.11]	0.44[0.38–0.60]	0.05*
	(CSP) Im-Only-Res	(Single-Phase) Com-Res	0.59[0.57–0.69]	0.67[0.63–0.70]	0.05*	0.63[0.48–1.11]	0.52[0.46–0.67]	0.05*
	(CSP) Im-Only-Res	(CSP) Ann-Com-Res	0.59[0.57–0.69]	0.76[0.70–0.79]	0.02*	0.63[0.48–1.11]	0.46[0.32–0.53]	0.02*
	(CSP) Ann-Res	(Single-Phase) Com-Res	0.74[0.65–0.79]	0.67[0.63–0.70]	0.38	0.44[0.38–0.60]	0.52[0.46–0.67]	0.81
	(CSP) Ann-Res	(CSP) Ann-Com-Res	0.74[0.65–0.79]	0.76[0.70–0.79]	0.05*	0.44[0.38–0.60]	0.46[0.32–0.53]	0.03*
	(Single-Phase) Com-Res	(CSP) Ann-Com-Res	0.67[0.63–0.70]	0.76[0.70–0.79]	0.08	0.52[0.46–0.67]	0.46[0.32–0.53]	0.22
Post–Stage2	(CSP) Im-Only-Res	(CSP) Ann-Res	0.70[0.69–0.72]	0.74[0.70–0.75]	0.22	0.72[0.70–0.90]	0.67[0.64–0.74]	0.38
	(CSP) Im-Only-Res	(Two-Phase) Com-Res	0.70[0.69–0.72]	0.70[0.69–0.75]	0.22	0.72[0.70–0.90]	0.60[0.58–0.65]	0.05*
	(CSP) Im-Only-Res	(CSP) Ann-Com-Res	0.70[0.69–0.72]	0.76[0.73–0.80]	0.02*	0.72[0.70–0.90]	0.42[0.41–0.45]	0.02*
	(CSP) Ann-Res	(Two-Phase) Com-Res	0.74[0.70–0.75]	0.70[0.69–0.75]	1.00	0.67[0.64–0.74]	0.60[0.58–0.65]	0.30
	(CSP) Ann-Res	(CSP) Ann-Com-Res	0.74[0.70–0.75]	0.76[0.73–0.80]	0.03*	0.67[0.64–0.74]	0.42[0.41–0.45]	0.02*
	(Two-Phase) Com-Res	(CSP) Ann-Com-Res	0.70[0.69–0.75]	0.76[0.73–0.80]	0.02*	0.60[0.58–0.65]	0.42[0.41–0.45]	0.02*
Post–Stage3	(CSP) Im-Only	(Two-Phase) Ann-Res	0.69[0.64–0.76]	0.79[0.78–0.80]	0.03*	0.98[0.74–1.54]	0.73[0.58–1.04]	0.38
	(CSP) Im-Only	(CSP) Com-Res	0.69[0.64–0.76]	0.80[0.78–0.82]	0.02*	0.98[0.74–1.54]	0.54[0.44–0.64]	0.02*
	(CSP) Im-Only	(Single-Phase) Ann-Com-Res	0.69[0.64–0.76]	0.84[0.83–0.85]	0.02*	0.98[0.74–1.54]	0.36[0.32–0.37]	0.02*
	(Two-Phase) Ann-Res	(CSP) Com-Res	0.79[0.78–0.80]	0.80[0.78–0.82]	0.30	0.73[0.58–1.04]	0.54[0.44–0.64]	0.02*
	(Two-Phase) Ann-Res	(Single-Phase) Ann-Com-Res	0.79[0.78–0.80]	0.84[0.83–0.85]	0.02*	0.73[0.58–1.04]	0.36[0.32–0.37]	0.02*
	(CSP) Com-Res	(Single-Phase) Ann-Com-Res	0.80[0.78–0.82]	0.84[0.83–0.85]	0.02*	0.54[0.44–0.64]	0.36[0.32–0.37]	0.02*
All Stages	(CSP) Im-Only	(CSP) Ann-Res	0.67[0.61–0.72]	0.77[0.73–0.81]	<0.001*	0.78[0.60–1.12]	0.60[0.44–0.74]	<0.001*
	(CSP) Im-Only	(Single-Phase) Com-Res	0.67[0.61–0.72]	0.78[0.68–0.81]	<0.001*	0.78[0.60–1.12]	0.52[0.36–0.63]	<0.001*
	(CSP) Im-Only	(CSP) Ann-Com-Res	0.67[0.61–0.72]	0.81[0.75–0.84]	<0.001*	0.78[0.60–1.12]	0.38[0.30–0.46]	<0.001*
	(CSP) Ann-Res	(Single-Phase) Com-Res	0.77[0.73–0.81]	0.78[0.68–0.81]	0.67	0.60[0.44–0.74]	0.52[0.36–0.63]	0.02*
	(CSP) Ann-Res	(CSP) Ann-Com-Res	0.77[0.73–0.81]	0.81[0.75–0.84]	<0.001*	0.60[0.44–0.74]	0.38[0.30–0.46]	<0.001*
	(Single-Phase) Com-Res	(CSP) Ann-Com-Res	0.78[0.68–0.81]	0.81[0.75–0.84]	<0.001*	0.52[0.36–0.63]	0.38[0.30–0.46]	<0.001*

The best ranked FCN input configurations across individual user inputs were compared. Different stages are presented along the larger row blocks. Metric values are displayed as median [IQR]. No additional user inputs (Im-Only), annular curve input (Ann), commissural landmarks input (Com) and resampling (Res). Significant p values (<0.05) marked with an asteroid (*). At individual stages of repair and all stages combined, the combination of annular curve input and commissural landmarks is significantly different from both the images only input and the annular curve only input. No significant difference could be found when comparing the annular curve only input and commissural landmarks only input.

For the merged leaflets, at all stages combined, significant differences (*p* < 0.05) could be found in almost all pairwise comparisons of the best ranked FCN variants. Notably, no significant difference could be found in DSC and MBD between annular curve input and the combined inputs of annular curve and commissural landmarks. Similarly, in individual stages, no significant difference could be found in DSC and MBD (except post-stage 2 repair) between annular curve input and the combined inputs of annular curve and commissural landmarks. Furthermore, no significant difference could be found for MBD and/or DSC between using images only and the input of commissural landmarks. At post-stage 1, the merged leaflets showed no significant difference for DSC between the input of annular curve and commissural landmarks.

For the individual leaflet averages in all stages combined, almost all pairwise comparisons showed significant differences. However, no significant difference could be found in DSC when comparing annular curve only input and commissural landmarks only input. In individual stages of repair, no significant difference could be found for DSC and MBD (pre-stage 1, post-stage 1, and post-stage 2) when comparing annular curve only input and commissural landmarks only input. In addition, no significant difference in DSC (post-stage 1 and post-stage 2) and MBD (post-stage 2 and post-stage 3) could be found between using images only and the annular curve input only. Finally, at post-stage 2 repair, no significant difference could be found for DSC between using images only and the input of commissural landmarks only. In all individual stages of repair (except post-stage 1) the commissural landmarks alongside with the annular curve performed superiorly compared to the annular curve alone or the commissural landmarks alone.

Generally, for both the merged leaflets and the individual leaflet averages, the annular curve input alone performed superiorly to the input of commissural landmarks alone. Also, additional user inputs always resulted in a higher DSC and a lower MBD. See Tables 6, 7 for more details.

### Examples of FCN Inference

**Figure 7:**
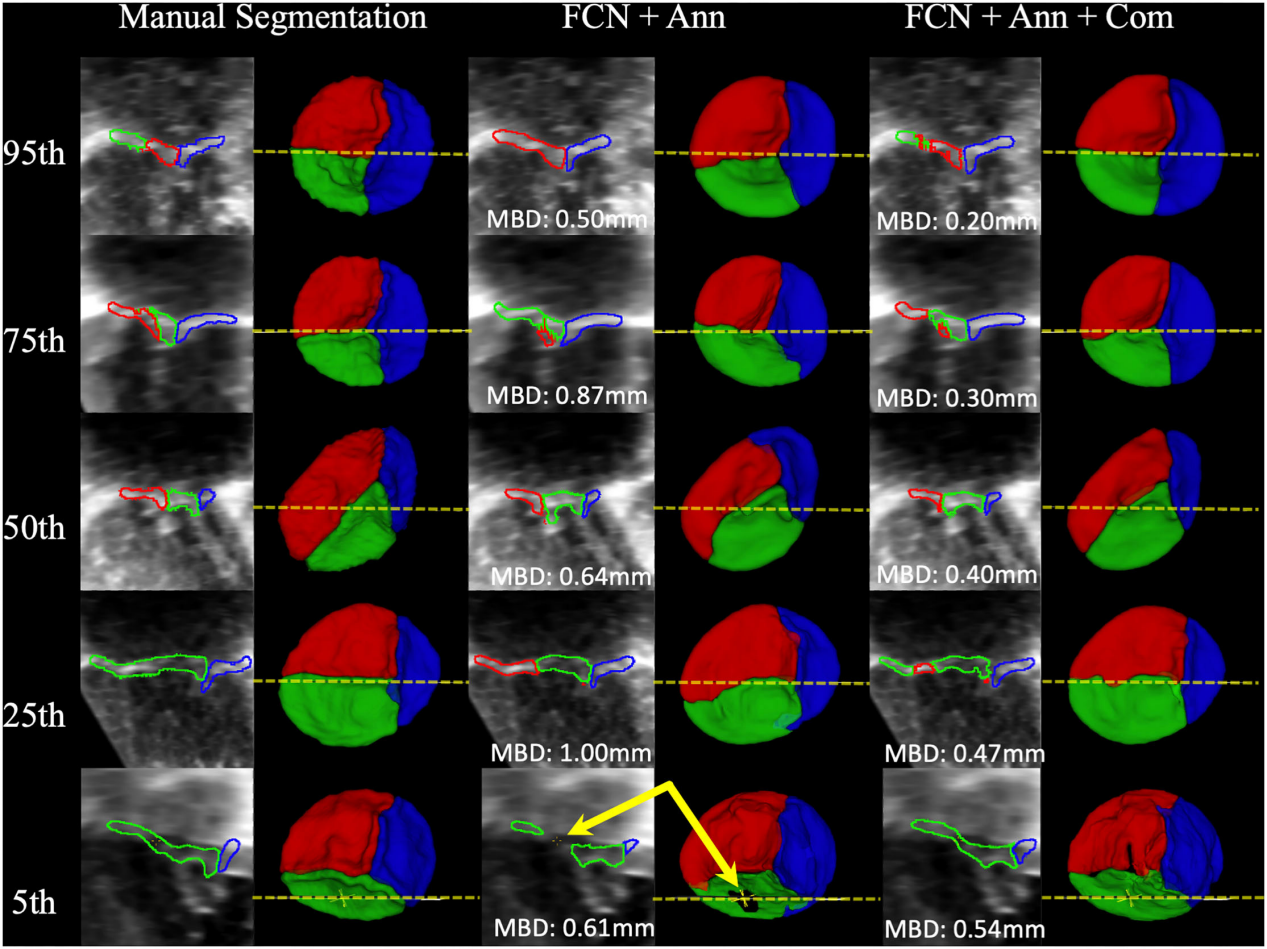
Examples of Segmentations predicted by the FCN. From **Left to Right**: 2D view of the MS frame (including MBD displayed at the bottom) and 3D segmentation model (ventricular view including indicator of 2D slice location) for Manual Segmentation, FCN + Annulus (Ann), and FCN + Ann + Commissures (Com). From **Top to Bottom**: 95th, 75th, 50th, 25th, and 5th percentile for MBD based on the best ranked CSP FCN with Ann + Com. The bottom row (5th percentile) shows an example of ultrasound signal dropout (indicated by arrows) that influenced the inferred segmentation using the annular curve only relative to the manual segmentation in which the user enforced the topology. When using the commissural landmarks in addition to the annular curve as FCN inputs, individual leaflet boundary detection improved and FCN segmentation was less sensitive to signal dropouts.

Example segmentations produced by FCN inference at the 95th, 75th, 50th, 25th, and 5th percentiles for MBD are shown in Figure 7. High-quality segmentations closely approximated the leaflet structure, and the overall configuration was correct down to the 5th percentile. However, in some cases, signal dropout in the image resulted in the FCN creating a hole in the leaflet, while the expert segmenter filled the hole due to externally derived knowledge of the valve function (review of 2D color Doppler images). The time to run scripted inference on a single dataset was under 6 seconds on a single GPU and approximately 30 seconds on a single CPU.

## Discussion

TV failure remains a critical factor in both the quality of life and survival of patients with HLHS (1). Rapid, accurate creation of valve models remains a roadblock to the application of quantitative modeling and simulation of the tricuspid valve in children with HLHS (6, 14, 16, 17). To the best of our knowledge, this is the first description of a DL-based approach to the segmentation of TV leaflets from 3DE, and to the segmentation of valves in children with congenital heart disease. Our work indicates that FCN-based segmentation may increase the clinical feasibility of 3DE-derived patient-specific modeling and quantification in HLHS and other challenging populations with both congenital and acquired valve disease.

Previous work on the segmentation of valve leaflets in 3DE has focused on TEE 3DE images of the mitral valve in adult patients (40–45). In particular, Pouch et al. demonstrated mitral valve segmentation by combining multi-atlas techniques with deformable models in both semi-automatic (18, 40) and fully-automatic workflows (41). Notably, the TEE probe's proximity to the mitral valve and excellent acoustic windows typically results in higher quality (higher signal to noise) than those acquired by TTE. However, at present, there is no 3D TEE probe suitable for children less than 20 kg. Therefore, TTE is often used to generate 3DE images in pediatric cases. Transthoracic images have the additional benefit of being able to be acquired without sedation as part of routine clinical evaluations in children and adults. However, children are often mobile during exams which makes it more challenging to acquire high quality images. Children are also less likely to cooperate for high temporal resolution, EKG-gated, breath held acquisitions. These factors may contribute to decreased image quality (decreased signal to noise, increased artifact) in TTE images relative to TEE images. Given the difficulty of the problem, there is relatively little experience in the literature concerning segmentation of individual valve leaflets from TTE, particularly in children and congenital heart disease (16).

Segmentation of individual valve leaflets from 3DE in general is also challenging due to relatively low contrast between the leaflets and blood and the lack of a clear boundary of the valve periphery (annulus) with the surrounding heart. Especially challenging is the identification of all three individual leaflet boundaries (anterior, posterior, and septal) in systole (valve closed; leaflets apposed). As a result, the majority of existing studies of the TV in HLHS have quantified the valve leaflets as a single merged unit which does not clearly evaluate the ability to separate individual leaflets (12, 13). Pouch et al. evaluated a semi-automated atlas and deformable model-based framework for the segmentation of TTE images of the TV in a handful of pediatric patients with HLHS (19). Notably, this previous work only describes the MBD for the merged leaflet structure, and not the individual leaflets. As such, the merged MBD metric forms the only basis for comparison between these studies and our current study. This comparison is provided in Table 8. For TV segmentation in HLHS subjects, Pouch et al. demonstrated segmentation with an MBD of 0.8 ± 0.2 mm relative to the TV ground truth expert segmentations. Our best current FCN approach (CSP, annular curve and commissural landmarks, with resampling) resulted in an average MBD of 0.34 ± 0.11 mm and a median MBD of 0.33 [0.26–0.4] mm, which outperforms this previously published technique. Furthermore, this FCN approach achieved a mean MBD of 0.38 ± 0.13 mm and a median MBD of 0.38 [0.3–0.46] mm for individual leaflets, as opposed to the merged valve. Our method also compares favorably to similar techniques applied to segmentation of the mitral valve from TEE, which may have benefited from the higher signal to noise typical of mitral valve TEE images relative to TTE images (Table 3).

**Table 8:** Comparison of MBD of the merged leaflet structure between previously published techniques and our current FCN approach.

	**MBD**
**Mitral**	
Pouch et al. (semi-automatic) (18)	1.3 ± 0.7
Pouch et al. (fully-automatic) (41)	0.6 ± 0.2
**Tricuspid**	
Pouch et al. (semi-automatic) (19)	0.8 ± 0.2
This FCN method (semi-automatic)	0.3 ± 0.1

There are other meaningful differences between the DL-based approach and the previously published multi-atlas/deformable model techniques. The multi-atlas-based approach can create a segmentation with only a few example atlases, while the DL-based approach demonstrated here used a much larger number of segmentations for training. Thus, in the situation where only a few atlases are available, the multi-atlas-based methods may be preferable. However, the atlases chosen are typically required to be highly representative of the valve structure to be segmented or risk biasing the segmentation.

Both atlas-based and DL-based methods require user interaction in the form of landmark placement to roughly register the images and initialize semi-automatic modeling. However, the semi-automatic atlas-based approach required the placement of 5 landmarks: an annular septal point, three commissural landmarks, and a leaflet centerpoint where the three leaflets come together. In our best DL-based technique, we provided the annular curve and commissural landmarks to the FCN. When comparing results for a merged valve, providing the annular curve alone was superior to using only the commissural landmarks as input. On the contrary, when comparing results for the average over separate individual leaflets, providing the commissures alone was superior to using only the annulus as input. It appears that the annular curve may inform restriction of the segmentation to the valve region, whereas the commissures help define the boundaries between individual leaflets.

Another difference between prior atlas-based modeling and our FCN-based modeling is that the atlas-based modeling was applied in concert with a deformable model. Application of a deformable model provides a basic structure and ensures leaflet topology even in the presence of signal dropout in the 3DE image. Unlike the previous atlas-based approaches, our FCN based method does not use any prior knowledge of leaflet topology. Signal dropouts pose a major challenge for valve segmentation algorithms involving 3DE datasets (46), as demonstrated in Figure 7. As such, signal dropout can result in holes in the FCN segmented leaflets. When manually segmenting the TV leaflets, a human user integrates prior knowledge of leaflet topology derived from 2D, 2D color, and 3D volume-rendered images of the TV, and may fill in dropout areas using this extraneous information. For example, color Doppler imaging both by 2D and 3D can determine the difference between dropout and a real leak (regurgitation). If there is no color Doppler signal in a dropout region, the human user knows that the “hole” represents a deficit of image quality, rather than an actual hole and a source of regurgitation. Notably, the addition of commissural landmarks as inputs to the FCN seemed to facilitate removal of false holes due to signal dropout (Figure 7). However, this was not effective in all cases. Application of a deformable template could be beneficial, but may also “overstep”, and fill holes that are actually present due to regurgitation. In the future, 2D and 3DE color Doppler information, which human observers utilize to reliably tell the difference, could be incorporated into the input data to improve erroneous interpretation of signal dropout.

Incorporation of a shape prior can bring atlas-like information into an FCN-based framework (23–25). Kervadec et al. tackle highly unbalanced segmentation by introducing a weighted loss using embedded shape information of ground truth segmentation distance maps (25). Medley et al. introduced a DL-based network for segmentation of the left ventricle (LV) in cardiac magnetic resonance images using landmarks of active shape models for object fitting (24). Oktay et al. proposed a framework for DL-based segmentation of the left ventricle (LV) in 3DE images using shape priors to follow global anatomical properties (23). The proposed framework consists of a two stage segmentation. First, an autoencoder (AE) is trained with ground truth segmentation masks to learn a latent representation embedding global information. Secondly, an FCN is trained optimizing toward the AE learned latent representation. In early experiments we trained an AE as introduced in (23), but segmentation quality did not improve and resulted in overly homogenized TV leaflet segmentations. One reason for this could be the large variability in shape of TV leaflets when compared to the relatively uniform shape of the LV. Finally, in comparison to DL-based approaches, atlas-based segmentation can be time consuming. The DL-based segmentation is performed in a fraction of the time needed for the atlas-based approach on GPU or more basic CPU hardware.

We explored multiple FCN variants, and the optimal configuration varied depending on the FCN inputs. FCN variants which incorporated user input performed better than those with user input alone. In FCN variants that utilize user inputs, the FCN variants with resampling generally outperformed no resampling. This trend extended to both all stages of repair combined and individual stages of repair.

For the merged leaflet models, no significant difference could be found between using the annular curve input and the combination of annular curve and commissural landmarks. The commissural landmarks are located along the annular curve to highlight the location at which leaflet separation occurs. As such, when evaluating the merged TV segmentation, the commissural landmarks may not add any additional utility given that precise leaflet separation that would be evaluated by the merged metrics, and any information they may provide for the border of the valve is redundant with the annular curve. In contrast, FCN variants with commissural landmarks show significant improvement when compared against the FCN variant with images only (and not annular curve input) suggesting that, in the absence of the annular curve, commissural landmarks may provide useful information to the FCN (e.g. provision of region of interest) in merged leaflets as well as individual leaflets.

Providing additional image frames other than the segmented MS frame was generally helpful for the more challenging task of segmentation of individual leaflets. The best performing CSP FCN input configuration provides consecutive phase frames, which may provide an indicator about the separation between leaflets as the leaflets open and close throughout the cardiac cycle. However, more frames are not always better; the Single-Phase or Two-Phase approach often performed better than the Four-Phase or the CSP approach which provided additional input frames.

The optimal FCN and performance also varied by stage of repair. As noted, the best performing network for the complete population was the FCN utilizing consecutive systolic phases (CSP) with resampling and input of annular curve and commissural points which provided a MBD of 0.38 [0.3–0.46] mm and a DSC of 0.81 [0.75–0.84]. For pre-stage 1 and post-stage 3 repair images, the highest ranked FCN variants performed best across all stages of repair with an average MBD of 0.21 [0.21–0.27] mm and 0.36 [0.32–0.37] mm, respectively. In contrast, the post-stage 1 and post stage 2 performed less than ideal with an MBD of 0.46 [0.32–0.53] mm and 0.42 [0.41–0.45] mm, respectively. Notably, the majority of our training images were post-stage 3 repair (57%) and pre-stage 1 repair (18%) which might be the reason for their better performance and for requiring less input image frames. In contrast, only 15% of our training images stem from post-stage 1 repair and only 9% of our training data originated from post-stage 2.

Further optimization of the FCN and the optimal input to the FCN is an important area for future work. In particular, the number of frames in a cardiac cycle depends on the acquisition's frame rate and the patient's heart rate. A higher 3DE frame rate may allow the FCN to resolve more frames during valve opening and closing, which may inform recognition of leaflet boundaries. Lastly, the current approach may be optimized by further exploration of shape-aware techniques (23–25).

## Deployment of Model

We are actively working to deploy this evolving model for open translational research use. To accomplish this, we plan on integrating pretrained models into our 3D Slicer segmentation workflow. On the client side, the user will provide minimal inputs (i.e., annular contour). Fully automatic pre-processing including reorientation, resampling and cropping will be applied, and associated with additional user inputs (e.g. commissural landmarks), which will then be sent to the remote server. The remote server will run inference and provide a rapid segmentation of the TV from 3DE images to the client application. Our current efforts are based on the MONAI framework (47) with networks and their implementation detailed at https://github.com/JolleyLabCHOP/DeepHeart.

## Limitations

The availability of 3DE images of TV in HLHS (training data) is relatively small compared to large adult populations. There is currently no extension of our approach to images of the mitral or tricuspid valves of normal children or adults as we do not currently have a large cohort of segmented tricuspid valve/image pairs in those populations, or the availability of 3DE images to segment.

In comparison of human segmentation intra/inter-user variability to DL-based segmentation, the trained FCN model received a cropped volume based upon the ground truth annular curve created by a human user. The human user neither received an annular curve nor a cropped volume for creating the TV segmentation.

## Conclusion

Accurate and efficient DL-based semi-automatic segmentation of the tricuspid valve in HLHS from 3DE images is feasible and results in an average accuracy on par with a single human observer, with less variability than that seen between two expert observers. The most accurate FCN configuration overall utilized consecutive systolic image phases with resampling as well as user annotated annular curve and commissural landmarks to inform individual leaflet separation. Further development may enable rapid TV modeling and quantification, which in turn may inform surgical planning in this complex population. However, signal dropout remains a challenge, and further development and optimization are needed.

## Data Availability Statement

All results are available within the article as supplied. The training data was obtained from echocardiograms annotated at the Children's Hospital of Philadelphia with approval from the Institutional Review Board. This data is generated as part of ongoing NIH-funded analyses of the association of tricuspid valve structure to function (NIH R01 HL153166). Upon completion and publication of these studies the datasets will be released in accordance with evolving guidelines for NIH-funded research. In the interim, requests for data can be submitted to the senior author. Requests to access the datasets should be directed to JOLLEYM@chop.edu.

## Ethics Statement

This study was approved by the Institutional Review Board at the Children's Hospital of Philadelphia under protocols IRB 16-012853 and IRB 16-013091. Written consent for data collection and use for publication was obtained for data collected as part IRB 16-013091. Retrospective data obtained as part of routine care was considered exempt as part of IRB 16-012853. Written informed consent to participate in this study was provided by the participants' legal guardian/next of kin.

## Author Contributions

CH, DP, MJ, AL, and PG contributed to study design and implementation of the machine learning model. HN, PD, MF, and AC segmented and annotated echocardiographic images. CH, DP, MJ, AL, HN, MF, and AC contributed to data analysis and manuscript preparation. All authors agree to be accountable for the content of the work. All authors contributed to the article and approved the submitted version.

## Funding

This work was supported by the Big Hearts to Little Hearts, NIH R01 HL153166, the Department of Anesthesia and Critical Care at the Children's Hospital of Philadelphia (CHOP), a CHOP Cardiac Center Innovation Grant, the Cora Topolewski Cardiac Research Fund at CHOP, the CHOP Frontier Program, and a CANARIE's Research Software Program. In addition, DP and PG were supported by IBIB NAC P41EB015902, and by grants from Philips Research. However, Philips Research did not participate or otherwise contribute to this work in any way. DP was also supported by NIH 1R56AG064027 and 1R01AG064027.

## Conflict of Interest

PG and DP received funding from Philips Research. The funder was not involved in the study design, collection, analysis, interpretation of data, the writing of this article, or the decision to submit it for publication. The remaining authors declare that the research was conducted in the absence of any commercial or financial relationships that could be construed as a potential conflict of interest.

## Publisher's Note

All claims expressed in this article are solely those of the authors and do not necessarily represent those of their affiliated organizations, or those of the publisher, the editors and the reviewers. Any product that may be evaluated in this article, or claim that may be made by its manufacturer, is not guaranteed or endorsed by the publisher.
