# Supplementary material for: Segmentation of Tricuspid Valve Leaflets From Transthoracic 3D Echocardiograms of Children With Hypoplastic Left Heart Syndrome Using Deep Learning
Source: Front Cardiovasc Med. 2021 Dec 9;8:735587. doi: 10.3389/fcvm.2021.735587 (PMC8696083; doi:10.3389/fcvm.2021.735587)
Supplement: Supplementary file 3 [file Data_Sheet_1.ZIP › Supplemental_Figure_Captions.docx]

**Figure S1 Dice Similarity Coefficient (DSC) in Pre-Stage 1 Repair for all Input Frame Combinations (Single-Phase, Two-Phase, Four-Phase and CSP) and all Binary Combinations of the Annular Curve Input (Ann), Commissural Landmarks Input (Com) and Resampling (Res).** Data is presented for individual leaflets (anterior, posterior, septal) and the individual leaflet average. The boxes represent median DSC with IQR. The whiskers present 1.5*IQR past the low and high quartiles. The diamonds indicate outliers.

**Figure S2 Dice Similarity Coefficient (DSC) in Post-Stage 1 Repair for all Input Frame Combinations (Single-Phase, Two-Phase, Four-Phase and CSP) and all Binary Combinations of the Annular Curve Input (Ann), Commissural Landmarks Input (Com) and Resampling (Res).** Data is presented for individual leaflets (anterior, posterior, septal) and the individual leaflet average. The boxes represent median DSC with IQR. The whiskers present 1.5*IQR past the low and high quartiles. The diamonds indicate outliers.

**Figure S3 Dice Similarity Coefficient (DSC) in Post-Stage 2 Repair for all Input Frame Combinations (Single-Phase, Two-Phase, Four-Phase and CSP) and all Binary Combinations of the Annular Curve Input (Ann), Commissural Landmarks Input (Com) and Resampling (Res).** Data is presented for individual leaflets (anterior, posterior, septal) and the individual leaflet average. The boxes represent median DSC with IQR. The whiskers present 1.5*IQR past the low and high quartiles. The diamonds indicate outliers.

**Figure S4 Dice Similarity Coefficient (DSC) in Post-Stage 3 Repair for all Input Frame Combinations (Single-Phase, Two-Phase, Four-Phase and CSP) and all Binary Combinations of the Annular Curve Input (Ann), Commissural Landmarks Input (Com) and Resampling (Res).** Data is presented for individual leaflets (anterior, posterior, septal) and the individual leaflet average. The boxes represent median DSC with IQR. The whiskers present 1.5*IQR past the low and high quartiles. The diamonds indicate outliers.

**Figure S5 Mean Boundary Distance (MBD) in Pre-Stage 1 Repair for all Input Frame Combinations (Single-Phase, Two-Phase, Four-Phase and CSP) and all Binary Combinations of the Annular Curve Input (Ann), Commissural Landmarks Input (Com) and Resampling (Res).** Data is presented for individual leaflets (anterior, posterior, septal) and the individual leaflet average. The boxes represent median MBD with IQR. The whiskers present 1.5*IQR past the low and high quartiles. The diamonds indicate outliers.

**Figure S6 Mean Boundary Distance (MBD) in Post-Stage 1 Repair for all Input Frame Combinations (Single-Phase, Two-Phase, Four-Phase and CSP) and all Binary Combinations of the Annular Curve Input (Ann), Commissural Landmarks Input (Com) and Resampling (Res).** Data is presented for individual leaflets (anterior, posterior, septal) and the individual leaflet average. The boxes represent median MBD with IQR. The whiskers present 1.5*IQR past the low and high quartiles. The diamonds indicate outliers.

**Figure S7 Mean Boundary Distance (MBD) in Post-Stage 2 Repair for all Input Frame Combinations (Single-Phase, Two-Phase, Four-Phase and CSP) and all Binary Combinations of the Annular Curve Input (Ann), Commissural Landmarks Input (Com) and Resampling (Res).** Data is presented for individual leaflets (anterior, posterior, septal) and the individual leaflet average. The boxes represent median MBD with IQR. The whiskers present 1.5*IQR past the low and high quartiles. The diamonds indicate outliers.

**Figure S8 Mean Boundary Distance (MBD) in Post-Stage 3 Repair for all Input Frame Combinations (Single-Phase, Two-Phase, Four-Phase and CSP) and all Binary Combinations of the Annular Curve Input (Ann), Commissural Landmarks Input (Com) and Resampling (Res).** Data is presented for individual leaflets (anterior, posterior, septal) and the individual leaflet average. The boxes represent median MBD with IQR. The whiskers present 1.5*IQR past the low and high quartiles. The diamonds indicate outliers.
